# Removal of Persistent Sulfamethoxazole and Carbamazepine from Water by Horseradish Peroxidase Encapsulated into Poly(Vinyl Chloride) Electrospun Fibers

**DOI:** 10.3390/ijms23010272

**Published:** 2021-12-27

**Authors:** Jakub Zdarta, Oliwia Degórska, Katarzyna Jankowska, Agnieszka Rybarczyk, Adam Piasecki, Filip Ciesielczyk, Teofil Jesionowski

**Affiliations:** 1 Institute of Chemical Technology and Engineering, Faculty of Chemical Technology, Poznan University of Technology, Berdychowo 4, PL-60965 Poznan, Poland; oliwia.degorska@gmail.com (O.D.); katjan@kt.dtu.dk (K.J.); aga.rybarczyk14@gmail.com (A.R.); filip.ciesielczyk@put.poznan.pl (F.C.); teofil.jesionowski@put.poznan.pl (T.J.); 2 Process and Systems Engineering Centre (PROSYS), Department of Chemical and Biochemical Engineering, Technical University of Denmark, Søltofts Plads, Building 227, DK-2800 Kongens Lyngby, Denmark; 3 Institute of Materials Engineering, Faculty of Materials Engineering and Technical Physics, Poznan University of Technology, Piotrowo 3, PL-60965 Poznan, Poland; adam.piasecki@put.poznan.pl

**Keywords:** electrospinning, enzyme immobilization, encapsulation, horseradish peroxidase, biodegradation, pharmaceuticals

## Abstract

Enzymatic conversion of pharmaceutically active ingredients (API), using immobilized enzymes should be considered as a promising industrial tool due to improved reusability and stability of the biocatalysts at harsh process conditions. Therefore, in this study horseradish peroxidase was immobilized into sodium alginate capsules and then trapped into poly(vinyl chloride) electrospun fibers to provide additional enzyme stabilization and protection against the negative effect of harsh process conditions. Due to encapsulation immobilization, 100% of immobilization yield was achieved leading to loading of 25 μg of enzyme in 1 mg of the support. Immobilized in such a way, enzyme showed over 80% activity retention. Further, only slight changes in kinetic parameters of free (*K_m_* = 1.54 mM) and immobilized horseradish peroxidase (*K_m_* = 1.83 mM) were noticed, indicating retention of high catalytic properties and high substrate affinity by encapsulated biocatalyst. Encapsulated horseradish peroxidase was tested in biodegradation of two frequently occurring in wastewater API, sulfamethoxazole (antibiotic) and carbamazepine (anticonvulsant). Over 80% of both pharmaceutics was removed by immobilized enzyme after 24 h of the process from the solution at a concentration of 1 mg/L, under optimal conditions, which were found to be pH 7, temperature 25 °C and 2 mM of H_2_O_2_. However, even from 10 mg/L solutions, it was possible to remove over 40% of both pharmaceuticals. Finally, the reusability and storage stability study of immobilized horseradish peroxidase showed retention of over 60% of initial activity after 20 days of storage at 4 °C and after 10 repeated catalytic cycles, indicating great practical application potential. By contrast, the free enzyme showed less than 20% of its initial activity after 20 days of storage and exhibited no recycling potential.

## 1. Introduction

The worrying increase of pharmaceutical concentrations in surface and groundwater may contribute to many diseases of animals and humans. Among pharmaceuticals, there are antibiotics the uncontrolled increase in the amount of which in the aquatic environment can lead to serious disruptions of living organisms’ health. One of them is sulfamethoxazole (SMX), which is a sulfonamide antibiotic, that can be used for the treatment of bacterial infections, such as diarrhea, coccidiosis, or gastroenteritis. Moreover, SMX combined with trimethoprim is widely used in curing pneumonia, which is a respiratory disease [1]. Due to the effective action against both gram negative and positive bacteria it is one of the most commonly used antibiotics in the animal and food industry. The extensive application of SMX has resulted in the common presence of this compound in different environments, for example in surface water, where SMX concentrations fluctuate from nano-gram per liter to micro-gram per liter [2]. These tremendous quantities can cause antibiotic resistance of bacteria and threaten human health.

Nevertheless, sulfamethoxazole is not the only pharmaceutical widely found in the aquatic environment. Carbamazepine (CBZ) is an anticonvulsant medication used mostly in the treatment of epilepsy and neuropathic pain. It can be successfully used in treating bipolar depression but also in trigeminal neuralgia treatment, where up to 10% of CBZ is excreted unreacted from the human body. Because of its high persistency, CBZ can be found in almost ten times higher concentrations than other micropollutants, reaching up to thousands of ng/L in groundwater, surface water and effluents, which can be removed through biological wastewater treatment with an average efficiency of around 30% [3]. The occurrence of this pharmaceutical may not be an immediate threat to aquatic ecosystems or human health, but effective removal is still important for safe water reuse applications and drinking water treatment [4].

Nowadays due to the high hydrophilicity and chemical stability carbamazepine cannot be successfully removed by traditional physicochemical remediation methods. Different methods that provide high removal efficiency can be used, nevertheless, each one of them has some disadvantages. Advanced oxidation processes can cause the production of even more toxic by-products and for membrane separation, the occurring problem is the utilization of concentrated retentates [5]. The same problem occurs for sulfamethoxazole, which cannot be removed by traditional methods. Nanofiltration, biodegradation by activated sludge or ion exchange are inefficient, where after 15 days more than 70% of the pharmaceutical can be detected in the effluents [6]. Taking into account the risks resulting from the uncontrolled release of pharmaceutically active substances into the environment, relatively low removal efficiencies of these compounds and their impact on living organisms, steps should be taken to remove them efficiently. One of the best alternatives for pharmaceuticals removal from wastewaters is enzymatic bioremediation, which can be more effective than recently used technologies. What is more, enzymatic treatment is eco-friendly, can be conducted under mild conditions, excluding also harmful and toxic solvents. Additionally, the problem of keeping alive the microorganisms from which the enzymes arrive is avoided and the potential of enzymes as biocatalysts can be easily exploited.

To enhance enzymes’ stability and their activity under different pH and temperatures, different immobilization methods are used, depending on process needs and enzyme properties [7]. Enzyme immobilization usually improves enzyme stability, reusability and operational ability. Enhancement of stability of biomolecules upon immobilization might be due to several factors including the formation of stable and/or multipoint enzyme-support bonds, limitation of enzyme rigidity due to its binding to the support, formation of internal interactions in the enzyme structure, prevention of enzymes from exposure to inactivation factors and partition of inhibitory compounds form enzyme surroundings [8,9]. Further, immobilization provides also suitable microenvironments for biomolecules by the generation of a hydrophilic or hydrophobic environment around enzyme molecules and ensures higher resistance towards harsh process conditions as support material acts as a heat or chemicals adsorber [10,11]. Thus, immobilization improves enzyme stability in both, quantitative and qualitative way making it possible to catalyze enzymatic conversions where free biocatalysts are not active. Further, properly performed and controlled enzyme immobilization reduces enzyme aggregation onto support and enzyme inhibition leading to higher process efficiency [12,13]. Moreover, immobilization protects the enzyme against conformational changes in its structure due to harsh process conditions leading to higher resistance to denaturation [14].

Currently, multimeric enzymes, involving dehydrogenases, oxidases, or catalases are one of the most widely used groups of enzymes. These are enzymes characterized by bigger molecular weight and bigger structure, as they are built of subunits. However, multimeric enzymes, due to the consistency of subunits are characterized by low operational stability and are very prone to inactivation due to subunits dissociation under harsh process conditions [15]. Further, besides enzyme inactivation, subunits dissociation could also lead to products contamination. The most effective approach to improve the stability and reusability of both monomeric and multimeric biomolecules is multipoint or multi-subunit immobilization. It is based on a multipoint attachment of the biomolecule or its subunits to the surface of support materials that is rich by the highly active chemical groups capable of covalent binding of the enzyme [16]. In this approach, the enzyme gained a high rigidity and remained almost unaltered during conformational rearrangements caused by heat, organic solvents, or pH [17]. It should be added that multipoint enzyme immobilization, besides stable enzyme binding to the support, reduces also subunits dissociation in consequence prolonging enzyme activity [18]. Further, multipoint immobilization is considered a technique that greatly stabilizes enzymes from different catalytic groups and is more effective than a simple one-point immobilization. Immobilization could also enhance enzyme activity, as well as its specificity and selectivity leading to wider practical application possibilities [19]. Finally, it should be noted that enzyme purification by immobilization could be also achieved, leading to a significant reduction in operational costs [20]. It should be added that immobilization usually creates diffusional limitations in the transport of substrate and products leading to a decrease in reaction efficiency. However, properly performed and controlled enzyme immobilization might reduce the creation of diffusional limitations. Further, the formation of diffusional limitation, observed usually in the case of porous support materials, might have a positive effect on enzyme activity, for instance in a situation where the substrate of the reaction acts as an enzyme inactivation agent [12].

Among others immobilization strategies, encapsulation should be mentioned. Using this method production of capsules consisting of a core and a shell is possible. The core may consist of a solid, liquid, or gas phase and is protected by one or more layers of an envelope consisting of a semi-permeable membrane or a polymer. For the production of semi-permeable membranes, the most popular are nylon or cellulose materials that allow obtaining microcapsules with a diameter of 10–100 μm. During the process, the enzyme-containing material can be easily separated from the reaction system. In addition, the enzyme retains its catalytic properties longer, because it has no direct contact with the reaction environment, and therefore, is not contaminated or deactivated by constituents of the reaction mixture [21]. As was presented in previously published studies, the biocatalytic systems containing encapsulated enzymes can be successfully used for pharmaceutical removal [22].

Therefore, in the presented work, it was decided to produce a novel biocatalytic system made of horseradish peroxidase immobilized into sodium alginate capsules and trapped into poly(vinyl chloride) electrospun fibers. To the best of our knowledge, this is a first attempt to encapsulate horseradish peroxidase twice and use the obtained system in the removal of pharmaceuticals. Enzyme encapsulated into electrospun PVC was characterized in terms of its storage stability and reusability. Moreover, the produced biosystem was next used for the removal of two pharmaceuticals: sulfamethoxazole and carbamazepine. The reactions were carried out at various process conditions, such as time, pharmaceutical concentration, temperature, pH, amount of immobilized enzyme and H_2_O_2_ concentration to achieve the highest removal efficiencies of pharmaceuticals.

## 2. Results and Discussion

### 2.1. Characterization of Electrospun Fibers before and after Enzyme Encapsulation

**Figure 1:**
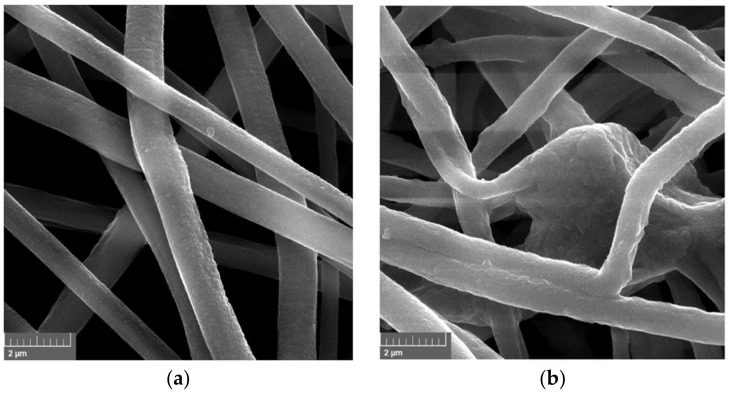
SEM photos of: (**a**) PVC fibers; (**b**) PVC fibers with encapsulated HRP.

The morphology of electrospun fibers made of PVC before and after enzyme encapsulation was evaluated based on SEM images (Figure 1). The produced electrospun fibers before HRP encapsulation are characterized by smooth structure and average diameters less than 1 μm, whereas material after enzyme encapsulation possesses thicker fibers, which diameters exceed 1.5 μm. The bulges of the fibers observed in Figure 1b, indicate the presence of enzymes inside the fiber. An increase of fiber diameters after enzyme encapsulation into electrospun fibers was also observed by dos Santos et al. [23], who showed that after xylanase encapsulation into poly(vinyl alcohol) material, the average fiber diameter increased from 257 nm to 449 nm. What is more, produced PVC electrospun fibers form a 3D open structure, that facilitates the easy circulation of substrates and products and creates the microenvironment suitable for catalytic reactions [24].

### 2.2. Characterization of the Biocatalytic System Produced

In the present study, horseradish peroxidase was immobilized by encapsulation. Prior to immobilization, the total enzyme amount used for immobilization was suspended in a polymer solution and was electrospun to produce polymeric fibers with enzyme inside. This approach leads to a 100% immobilization yield that corresponded to high enzyme loading of 25 μg of HRP into 1 mg of electrospun material (Table 1). Moreover, this approach gives an opportunity to control the amount of immobilized enzymes to avoid enzyme overloading and to produce systems with high activity. In our study over 80% activity retention by encapsulated HRP was noticed. The slight decrease of enzyme catalytic activity upon immobilization might be explained by the formation of steric hindrances around HRP active sites and partial blocking of the substrate diffusion due to surrounding support layers of sodium alginate and PVC [25]. On the other hand, such high activity retention might be explained by the high porosity of electrospun fibers that ensure access of substrates to enzymes active sites as well as characteristic of the encapsulation technique. In this approach enzyme is retained in the matrix mainly by physical interactions, therefore, interference in the structure of the biomolecule is limited, maintaining its high catalytic activity.

As horseradish peroxidase was immobilized by encapsulation into electrospun fibers, it is crucial to determine changes in enzyme-substrate affinity and reaction rate upon immobilization. Free HRP showed a Michaelis–Menten constant (*K_m_*) of 1.54 mM and a maximum reaction rate (*V_max_*) of 422 U/mg. By contrast, about 20% lower HRP affinity to the substrate (an increase of *K_m_* to 1.84 mM) followed by over 20% lower maximum reaction rate (*V_max_* decreased to 312 U/mg) was noticed for encapsulated HRP. This is directly related to the method of immobilization. In encapsulation, HRP is surrounded by the electrospun materials and additionally by the sodium alginate capsules that lead to partial blocking of the enzyme active sites, which reduced their accessibility to binding the substrate molecules [26]. Further, due to the external polymer layer also some diffusional limitations in the transport of substrate and products might have occurred. Nevertheless, only 20% lower *V_max_* noticed for immobilized enzymes was achieved mainly due to the highly porous structure of the electrospun material that facilitates efficient substrate supply [24]. Similar observations were made by Dai et al., who fabricated electrospun material from poly(D,L-lactide-*co*-glycolide) with encapsulated laccase and noticed only around 15% increase of *K_m_* mainly due to the high porosity of the electrospun fibers [27].

### 2.3. Stability Study of the Biocatalytic System Produced

**Figure 2:**
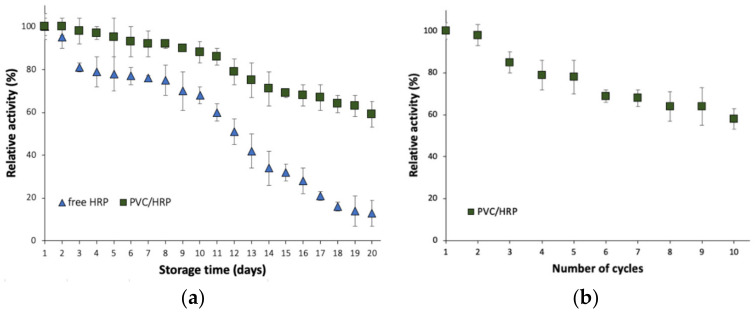
(**a**) Storage stability of the free and immobilized HRP; (**b**) reusability of the PVC/HRP-based biosystem.

Immobilization is one the most promising approaches to improve enzyme stability, operationality, as well as reusability. From a practical application point of view for the removal of micropollutants, obtaining a biocatalyst that is reusable and stable over a long period of time is of particular interest as these features might significantly affect operational costs of wastewater treatment. The HRP encapsulated into PVC electrospun fibers retained over 60% of its initial catalytic properties after 20 days of storage at 4 °C which is around 40% higher as compared to free HRP, which showed around 20% relative activity (Figure 2a). This corresponds also to a significant improvement of enzyme inactivation constant (*k_D_*), which decreased from 0.1065 1/day for the free enzyme to 0.0291 1/day for encapsulated HRP. Consequently, almost four times higher enzyme half-life time (*t_1/2_*) was reported for the immobilized enzyme, which reached 23.8 days. Further, obtained data indicate also the great reusability of the produced biocatalytic systems, as even after 10 repeated cycles over 60% of initial activity was retained (Figure 2b).

The significant improvement of storage stability and providing the opportunity to reuse the enzyme is directly related to enzyme deposition into the electrospun matrix and alginate capsules. This solution stabilizes the enzyme structure, providing a protective effect for the support materials against inactivation due to harsh process conditions, as well as providing a suitable microenvironment for high enzyme activity [28]. Nevertheless, the observed decrease in enzyme stability and reusability is probably related to partial enzyme elution from the support, as well as enzyme inhibition and inactivation over storage time and repeated use. In another study, Patel et al. also reported a protective effect of electrospun porous silica fibers on horseradish peroxidase structure and properties. Enzyme immobilized in that study showed around 90% of initial activity after five repeated catalytic cycles [26].

### 2.4. Removal of Sulfamethoxazole and Carbamazepine by the Inactivated HRP

As electrospun materials are characterized by high porosity and well-developed surface area, they may also show high sorption capacity towards pharmaceuticals. Therefore, prior to biodegradation experiments of the SMX and CBZ also sorption capacity of PVC electrospun fibers, using a system with inactivated encapsulated horseradish peroxidase was determined. For this purpose, PVC with thermally inactivated HRP was placed into 5 mg/L solutions of SMX and CBZ, separately. Obtained results showed that sorption efficiencies were less than 6% for both tested pharmaceuticals. As error values of these measurements in the study were estimated to be 5%, it can be concluded that sorption of the pharmaceuticals onto PVC fibers was negligible and there is rather a relation to the catalytic conversion by immobilized HRP.

### 2.5. Removal of Sulfamethoxazole by the Immobilized HRP

**Figure 3:**
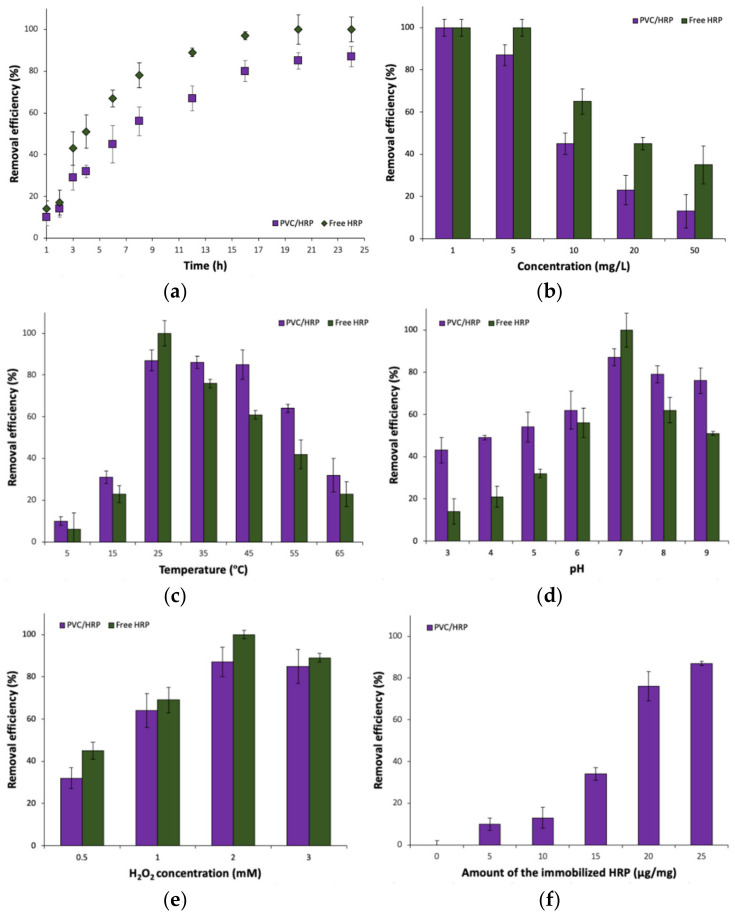
The effect of: (**a**) time process; (**b**) concentration of SMX; (**c**) temperature; (**d**) pH; (**e**) H_2_O_2_ concentration and (**f**) amount of the immobilized HRP on SMX removal efficiency.

The removal efficiency of sulfamethoxazole from 5 mg/L solutions, by free and encapsulated horseradish peroxidase, was investigated for 24 h (Figure 3a). The longer the process was conducted, the higher values of degradation of the antibiotic were obtained. After 6 h of the process, the efficiency of pharmaceutical removal equaled 45% and after 12 h the antibiotic was removed almost in 70%. Total removal of SMX after 24 h reached 85% indicating that encapsulated enzyme is well protected by both, sodium alginate and PVC shell against process conditions and retained its high activity over whole process duration. By contrast, biodegradation of SMX by free enzyme reached 100% after 20 h of the process, clearly showing good suitability of the HRP for sulfamethoxazole removal.

The influence of SMX concentration (Figure 3b) on its removal efficiency was also investigated, where five concentrations were taken into account: 1, 5, 10, 20 and 50 mg/L. Presented data clearly indicate that the higher concentration of antibiotics the lower its removal efficiency from the solution by both, free and immobilized HRP. This could be explained in two ways. The first one is inhibition of the horseradish peroxidase enzyme active sites by substrate molecules at higher concentrations, whereas in the case of the immobilized enzyme, the reason might be an insufficient amount of encapsulated biocatalyst used for removal of SMX from solutions at higher concentrations [29]. Both, free and immobilized HRP showed similar removal profiles over tested SMX solutions, however, the free enzyme exhibited over 20% higher removal efficiency. For 1 mg/L solution of SMX, 100% of degradation rate was obtained—that was the highest value among all tested concentrations. For encapsulated HRP, and using a 5 mg/L solution of SMX, a drop in the efficiency was insignificant and the removal rate reached around 85%. Radical decrease of degradation efficiency was observed for 10, 20 and 50 mg/L solutions for which the removal efficiency reached around 40%, 20% and 15%, respectively.

The effect of temperature on SMX removal was also investigated (Figure 3c). The efficiency of removal increased gradually with an increase of temperature and picked at a temperature of 25 °C, where 100% and 85% of degradation efficiency using free and encapsulated HRP respectively, was obtained. For the temperatures 35 °C and 45 °C, the efficiency remained stable and fluctuated slightly indicating the high stability of encapsulated HRP against thermal inactivation due to stabilization and protection of the enzyme structure. Above that temperature the drop of the degradation rate is visible and for the temperature of 55 °C, the efficiency equaled to 70%. However, the lowest biodegradation value was noticed for the temperature of 65 °C where the efficiency of SMX removal was at the level of 30%. By contrast, free enzymes showed higher activity at optimal temperature (25 °C), however, even slight changes in temperature led to lower removal rates. The rapid drop of degradation efficiency can be related to the partial denaturation of the enzyme due to the higher temperature of the process that lowers its total activity in this process. On the other hand, low removal efficiencies of SMX at temperatures 5 °C and 15 °C can be related to insufficient enzyme activation due to too low temperature [30]. Weng et al. used laccase with ABTS mediator for the removal of sulfonamide antibiotics. The most suitable temperature was 20 °C where around 90% of the antibiotic was removed. Above that temperature, the effectiveness drops to 70% at temperature 60 °C [31]. In the other study, Rahmani et al. investigated the influence of temperature on SMX removal by laccase. From 20 °C to 60 °C the drop of pharmaceutical degradation was inconsiderable, however, at the temperature of 70 °C and 80 °C significant reductions in process efficiency were observed [32].

The effect of pH was also a part of research to determine the most convenient removal conditions (Figure 3d). The lowest removal efficiency, equaled 43%, was obtained at the lowest pH 3, whereas 15% higher values were noted for pH 5 using encapsulated enzyme. The most suitable pH for free and immobilized horseradish peroxidase turned out to be pH 7, at which 100% and 85% of the antibiotic respectively, was removed from the solution. Above that value (at pH 9), the efficiency decreased, reaching around 70% and 50% for free and encapsulated HRP, respectively. The decrease can be related to the denaturation of the enzyme due to too high of a pH in the solution. Despite the protective effect of the carrier on the immobilized enzyme, degradation of the enzyme may occur, which was also presented by Rahmani et al. Laccase immobilized on silica beads was used for SMX removal over a wide pH range. Above pH 5 the relative activity of the enzyme slowly dropped reaching less than 40% at pH 9 [32].

The next investigated factor, that may affect the efficiency of antibiotic degradation, was the concentration of H_2_O_2_, as hydrogen peroxide acts as a co-substrate for horseradish peroxidase (Figure 3e) [33]. Over the whole analyzed H_2_O_2_ concentration range, free enzymes showed higher removal rates than immobilized HRP. Application of H_2_O_2_ solution 0.5 mM in concentration, caused the efficiency to be lower than 35%, however, when the concentration was increased up to 1 mM, the efficiency increased to 65% using the system after immobilization. At the same hydrogen peroxide concentration, free enzyme removed over 50% and over 70% of SMX, respectively. The highest removal efficiency of SMX was obtained when 2 mM H_2_O_2_ solution was used, in which the degradation of SMX reached around 85% and 100% for free and immobilized HRP, respectively. At the highest hydrogen peroxide concentration (3 mM), a decrease in the removal efficiency could be observed. This might be due to partial inhibition and inactivation of horseradish peroxides at higher peroxide concentrations [34]. It should be highlighted that HRP is sensitive towards high hydrogen peroxide levels and undergoes inactivation after long-term contact with H_2_O_2_ [35]. The horseradish peroxidase inactivation by the H_2_O_2_ might occur due to two-side attack of the hydrogen peroxide on the HRP molecule. At lower peroxide concentration the attack occurs from the heme site and the active site is not directly changed however, it might be inhibited by oxidized substrate [36]. At a higher hydrogen peroxide concentration, the oxidative effect of H_2_O_2_ is clearly seen. This results in partial oxidation of various regions of protein leading to irreversible changes in secondary and tertiary enzyme structure [37]. Therefore, control of H_2_O_2_ supply for both, free and immobilized HRP, is crucial during the bioremediation process in the presence of this enzyme. On the one hand, the enzyme could not show its highest activity due to too low of a peroxide concentration, on the other hand too high of an H_2_O_2_ concentration led to enzyme inactivation [38]. The obtained results can be compared with other work, where horseradish peroxidase was immobilized onto polyamide 6 fibers and applied for removal of dyes from aqueous solutions. It was shown that the removal efficiencies of Reactive Black 5 and Malachite Green increased with increase concentration of H_2_O_2_ finally reaching over 70% in the presence of 1 mM of H_2_O_2_ [39].

Furthermore, it can be seen from Figure 3f that the amount of enzyme used for pharmaceutical degradation plays a key role in the high biodegradation rate. Increasing the amount of the enzyme, increasing the efficiency of SMX removal. For 15 µg/mg of immobilized HRP, degradation of pharmaceutical was around 35%, whereas for 20 µg/mg the efficiency rapidly increased to about 80% and reached its maximum (around 85%) with 25 µg/mg of immobilized enzyme.

### 2.6. Removal of Carbamazepine by the Immobilized HRP

**Figure 4:**
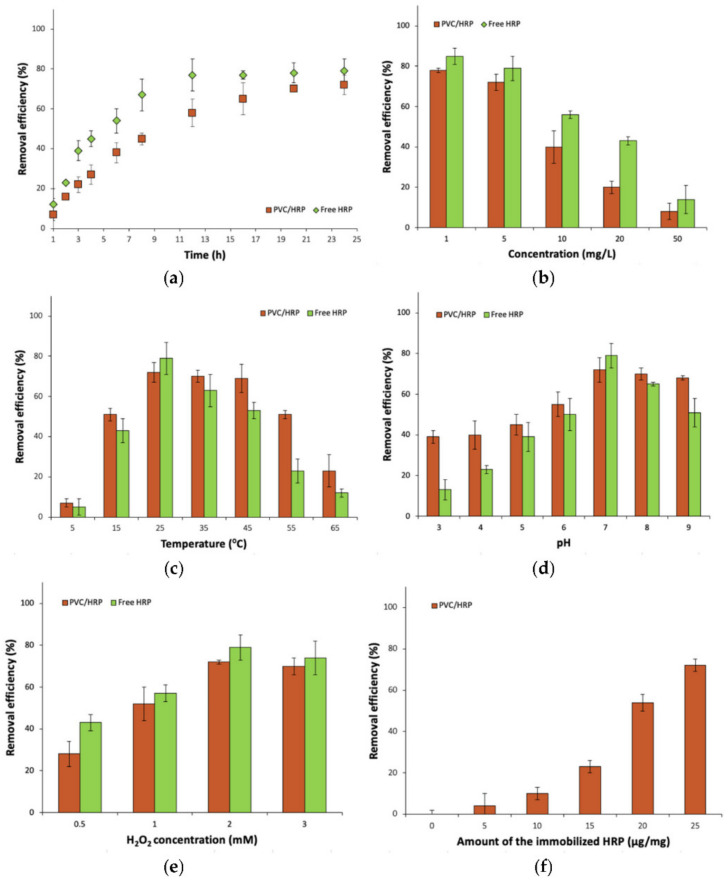
The effect of: (**a**) time process; (**b**) concentration of CBZ; (**c**) temperature; (**d**) pH; (**e**) H_2_O_2_ concentration and (**f**) amount of the immobilized HRP on CBZ removal efficiency.

Despite the removal of antibiotics, such as sulfamethoxazole, it was decided to investigate the degradation of carbamazepine–an anticonvulsant pharmaceutical–in various process conditions by producing a biosystem made of HRP encapsulated into PVC electrospun fibers and for the free enzyme, for comparison (Figure 4). The removal of these two compounds was due to the fact that they differ from each other not only by pharmaceutical action and chemical structure but also are known by different toxicity and their presence at various amounts in wastewater. It is also important to test produced immobilized HRP for removal of various compounds, as this enzyme, dependent on substrate type, catalyzes the oxidation reaction by oxidative coupling, selective hydroxylation, or N- and O-dealkylation, which in turn affects the conversion efficiency of substrates [40,41].

As compared to the results obtained during removal of SMX by free and encapsulated HRP, similar trends could be observed analyzing removal efficiency of carbamazepine, for each of investigated process parameters. The highest removal efficiency (84%) of CBZ by free enzyme was attained from 1 mg/L CBZ solution at 25 °C, pH 7, in the presence of 2 mM of H_2_O_2_. By contrast, the highest CBZ removal rate by encapsulated HRP, equaled 73%, was obtained after a 24 h reaction, from a pharmaceutical solution at a concentration of 1 mg/L, 25 °C, pH 7, using 25 µg/mg of the immobilized HRP and 2 mM of H_2_O_2_. However, the removal efficiency of CBZ is around 10% lower for free and immobilized HRP, than the degradation efficiency of SMX, which indicates a different mechanism of oxidation of these pharmaceuticals by HRP. In the case of carbamazepine, usually hydroxylation or epoxidation of the diazepine ring occurs, leading to the formation of the conversion products at similar mass and structure [21]. By contrast, in the case of sulfamethoxazole, catalytic oxidation leads to the breaking of the C–S and N–S bonds in the SMX structure. As a consequence final products at lower molecular weight are formed leading to their lower enzyme inhibition [42]. However, the examination of the products of the biocatalytic conversion of SMX and CBZ was not the major goal of this study. Another reason for the higher removal rate of SMX is better availability of the SMX reactive chemical groups for the enzyme, which facilitates the conversion by HRP, whereas in the case of CBZ, aromatic rings are conjugated, which makes it more challenging for the enzyme to oxidize this pharmaceutical.

It should be also stated that the removal of anticonvulsant pharmaceuticals by horseradish peroxidase is poorly described in the literature. However, one of the most interesting works in terms of the application of this oxidoreductase for CBZ removal was presented by Pylypchuk et al. [22]. They immobilized HRP onto magnetite particles and after that covered this biosystem using a silica matrix. Around 60% removal efficiency of CBZ by encapsulated HRP was obtained, which is lower compared to the result presented in this work. The low removal rate of CBZ was explained by the inefficient amount of pores in the silica layer, which are responsible for a constant flow of substrates and bioconversion products from and to the active center of this oxidoreductase.

The presented results decidedly show that the proposed biosystem made of HRP encapsulated into PVC fibers can be effectively used for the removal of pharmaceuticals from water solutions at various process conditions. Although free HRP showed 100% removal of SMX and over 75% removal of CBZ—higher than immobilized one, encapsulated HRP exhibited a higher removal rate over wider process conditions and showed relatively high reusability. Moreover, around 80% degradation efficiency of both used pharmaceuticals showed that the HRP immobilization could find application in removal of compounds with various chemical structures with relatively high efficiency.

## 3. Materials and Methods

### 3.1. Chemicals and Materials

Poly(vinyl chloride) (PVC, average M_w_ = 80.000 g/mol), tetrahydrofuran (THF), *N*,*N*-dimethylformamide (DMF), sodium alginate (SA), Pluronic**^®^** P-123, peroxidase from horseradish (HRP, activity of 150 U/mg), sulfamethoxazole (SMX), carbamazepine (CBZ), hydrogen peroxide (H_2_O_2_, 30%), Bradford reagent, 2,2′-azino-bis(3-ehylbenzothiazoline-6-sulfonic acid) diammonium salt (ABTS), sodium acetate and phosphate buffer solutions, Tris-hydrochloride and hydrochloric acid (HCl, 37%) were all purchased from Sigma-Aldrich (St. Louis, MO, USA).

### 3.2. Encapsulation of Horseradish Peroxidase into Electrospun Fibers

**Figure 5:**
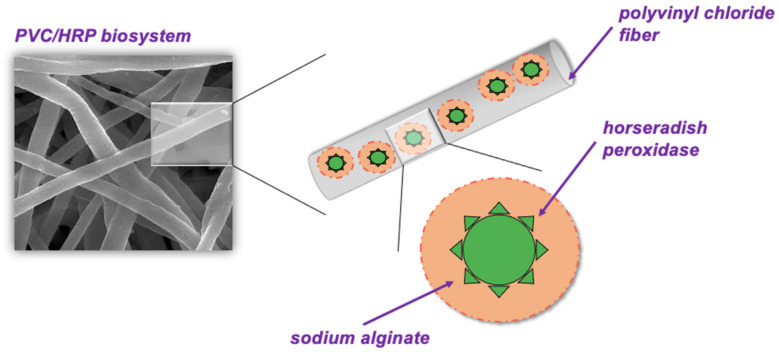
The scheme of electrospun PVC with encapsulated HRP.

PVC was dissolved in 3 mL THF:DMF solution (solvents volume ratio 1:1) to obtain a 12% (*w/v*) polymeric solution and next it was mixed for 24 h. After mixing 200 μL of the nonionic surfactant Pluronic**^®^** P-123 was added dropwise and mixed for another 1 h. At the same time, sodium alginate was dissolved in water for 24 h in order to obtain a 3% of SA solution. HRP was added to phosphate buffer solution at pH 7 to obtain solutions at concentration 1–5 mg/mL and next mixed for 3 h. After that SA solution was added to HRP solution to obtain HRP/SA mixture with volume ratio 1:1. Finally, 100 μL of HRP/SA solution was added to the PVC solution and mixed for 3 h. After mixing, the PVC/HRP final solution was placed in a syringe. The electrospinning process was carried out under applied voltage of 13.7 kV, feed rate of 1 mL/h, for 30 min using homemade electrospinning equipment keeping the distance between the nozzle and the collector as 15 cm. The electrospun fibers with HRP encapsulated at various concentrations were collected on the aluminum foil-covered steel collector and dried for 24 h at 25 °C. The scheme of obtaining electrospun PVC fibers with encapsulated HRP is presented in Figure 5. The encapsulation of HRP, additionally inside sodium alginate, was dictated by the fact that enzyme directly placed in the PVC solution could be inactivated by the presence of organic solvents as THF and DMF are used in the electrospinning process, which may lead to enzyme denaturation and changes in the structure of biomolecule [43]. Therefore, sodium alginate—a natural polysaccharide, due to its biocompatibility and stability at harsh conditions, can provide additional protection for HRP during electrospinning of fibers with enzymes [44].

### 3.3. Kinetic Parameters of the Free and Immobilized Horseradish Peroxidase

The catalytic activity of HRP encapsulated into PVC fibers was measured based on the model reaction with ABTS. The PVC/HRP electrospun fibers with 5 mg of encapsulated and free HRP, as a reference sample, were placed in 5 mL of ABTS solution at a concentration of 0.1 mM. Experiments were conducted at pH 7 in 25 °C for 1 h. The relative activity of encapsulated and free horseradish peroxidase was determined using spectroscopic measurements at 420 nm (UV-750 spectrophotometer, Jasco, Tokyo, Japan). The concentration of ABTS after the oxidation process was calculated based on ABTS standard curve. 1 U of HRP corresponds to the amount of enzyme which oxidizes 1 μmol ABTS per minute under optimal process conditions. Based on the results, the relative activity (*RA*) of immobilized HRP was calculated using the following Equation (1):

(1) $$RA\left(\%\right)=\frac{IM_{A}}{I_{A}}\cdot 100\%$$

where *IM_A_* and *I_A_* denote the activity of immobilized enzyme and the activity of free enzyme, respectively.

Determination of the kinetic parameters, which were the Michaelis–Menten constant (*K_m_*) and maximum reaction rate (*V_max_*) of free and encapsulated HRP, was carried out using the above-described model reaction of ABTS oxidation performed under optimal process conditions with ABTS concentration ranging from 0.1 to 5 mM. The kinetic parameters were calculated based on the Hanes–Wolf plot.

### 3.4. Storage Stability and Reusability

The storage stability of free and encapsulated HRP stored in phosphate buffer at pH 7 at 4 °C was determined over 20 days based on spectrophotometric measurements according to model ABTS oxidation reaction under optimal process conditions (pH 7, 25 °C) using 5 mg of free or immobilized HRP. 100% of relative activity has been defined as the initial activity of free and immobilized enzymes. The reusability of the encapsulated horseradish peroxidase was determined in 10 repeated reaction cycles using model ABTS oxidation reaction under optimal process conditions. After each reaction step, electrospun fibers with immobilized HRP were separated from the post-reaction mixture, washed three times by phosphate buffer at pH 7 and placed into a fresh substrate solution. The activity of HRP in the first reaction cycle was defined as 100% activity. Inactivation constant (*k_d_*), and half-life (*t_1/2_*) of free and immobilized HRP were calculated based on the linear regression slope from the data of storage stability of free and encapsulated horseradish peroxidase. All measurements were made in triplicate and error bars are presented as means ± standard deviation.

### 3.5. Removal of Pharmaceuticals

To determine the sorption properties of PVC electrospun fibers with encapsulated HRP, prior to SMX and CBZ removal, the experiments were carried out using a biocatalytic system with a thermally inactivated enzyme. The PVC electrospun fibers with encapsulated horseradish peroxidase were placed for 4 h in a dryer at 80 °C, to deactivate the oxidoreductase. Next, electrospun fibers with the inactivated enzyme were placed in the vials with SMX and CBZ solution at a concentration of 5 mg/L (pH 7, 25 °C) for 24 h, separately. After a specified period of time, the absorbances of the solutions were measured and the sorption capacity of electrospun fibers with deactivated enzyme was determined.

**Figure 6:**
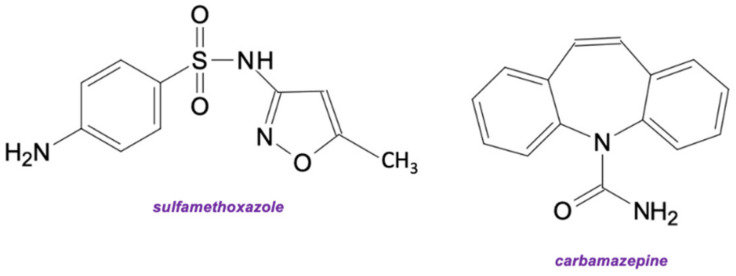
The structures of sulfamethoxazole and carbamazepine—pharmaceuticals used in the presented study.

The removal experiments of both pharmaceuticals were carried out under various conditions to study the effect of time, pharmaceutical concentration, temperature, pH, amount of immobilized biocatalyst and concentration of H_2_O_2_ on SMX and CBZ (Figure 6) removal efficiencies. In case of the effect of process duration, measurements points were at 1, 2, 3, 4, 6, 8, 12, 16, 20 and 24 h (pharmaceutical concentration 5 mg/L, 25 °C, pH 7, amount of immobilized HRP 25 μg/mg and H_2_O_2_ concentration 2 mM). The effect of SMX and CBZ concentration was measured for the pharmaceutical concentrations of 1, 5, 10, 20 and 50 mg/L (24 h, 25 °C, pH 7, amount of immobilized HRP 25 μg/mg and H_2_O_2_ concentration 2 mM). The experiments concerning the effect of temperature on SMX and CBZ removal efficiencies were carried out at temperature range 5–65 °C (measurement point every 10 °C) (24 h, pharmaceutical concentration 5 mg/L, pH 7, amount of immobilized HRP 25 μg/mg and H_2_O_2_ concentration 2 mM). In the case of measuring the effect of pH, measurement points were at pH range 3–9 (24 h, pharmaceutical concentration 5 mg/L, 25 °C, amount of immobilized HRP 25 μg/mg and H_2_O_2_ concentration 2 mM). The effect of the concentration of H_2_O_2_ was investigated using 0.5, 1.0, 2.0 and 3.0 mM of hydrogen peroxide solutions (24 h, pharmaceutical concentration 5 mg/L, 25 °C, pH 7 and amount of immobilized HRP 25 μg/mg). Whereas in the case of the effect of the amount of immobilized HRP, various amounts of HRP encapsulated into PVC fibers were used, ranging between 5 and 25 μg/mg (24 h, pharmaceutical concentration 5 mg/L, 25 °C, pH 7 and H_2_O_2_ concentration 2 mM). The measurements concerning the effect of contact time, pharmaceutical concentration, temperature, pH and concentration of H_2_O_2_ on SMX and CBZ removal efficiencies, were also conducted with the corresponding amount of free form of HRP. All measurements were made in triplicate and error bars are presented as means ± standard deviation.

### 3.6. Analytical Techniques

The morphology of the pristine PVC electrospun fibers and PVC fibers with encapsulated enzyme was observed using an EVO40 scanning electron microscope (SEM, Zeiss, Jena, Germany).

The results obtained from spectrophotometric measurements obtained using V-750 UV-Vis spectrophotometer (Jasco, Tokyo, Japan) allowed us to evaluate the changes in the concentration of the ABTS (λ = 420 nm), SMX (λ = 257 nm) and CBZ (λ = 284) during conversion processes. The concentrations of SMX and CBZ were determined using a calibration curve for each compound. The pharmaceuticals removal efficiency (*RE* (%)) was determined by using the following Equation (2):

(2) $$RE\left(\%\right)=\frac{C_{B}-C_{A}}{C_{B}}\cdot 100\%$$

where *C_B_* and *C_A_* denote pharmaceutical concentration before and after removal, respectively.

## 4. Conclusions

In the presented study we have demonstrated the novel approach for the encapsulation of horseradish peroxidase into sodium alginate capsules and deposition of such capsules into poly(vinyl chloride) electrospun fibers. The produced system showed retention of over 80% activity by encapsulated HRP, whereas slight changes in kinetic parameters of the enzyme upon immobilization indicated high substrate affinity and maximum reaction rate. The presented solution also improved the stability and reusability of the immobilized HRP, which are the key features from the practical application point of view. Over 60% of initial enzyme activity was retained after 20 days of storage and 10 repeated catalytic cycles. This makes obtained biocatalytic systems a promising tool for the removal of pharmaceutically active compounds, including sulfamethoxazole and carbamazepine. The high biodegradation potential of encapsulated HRP was proved by the obtained data, which shows that after 24 h of the process at pH 7 and temperature of 25 °C over 80% of both pharmaceuticals were removed from the 1 mg/L concentration solution in the presence of 2 mM H_2_O_2_. Therefore, it might be concluded that the presented data underline the great potential of the HRP encapsulated into poly(vinyl chloride) electrospun fibers in bioconversion of pharmaceuticals. Moreover, presented results also indicate the practical application potential of the produced systems in the removal of various micropollutants in wastewater treatment plants. Nevertheless, future studies are still highly required to transfer the proposed solution into a larger scale and to develop a solution for the continuous use of produced systems.

## Figures and Tables

**Table 1:** Amount of immobilized enzymes, immobilization yield, activity retention and kinetic parameters of the free and immobilized HRP.

Biocatalyst	Encapsulated HRP	Free HRP
**Amount of immobilized enzyme (μg/mg)**	25 ± 1	-
**Immobilization yield (%)**	100 ± 3	-
**Activity retention (%)**	81 ± 7	100 ± 2
***K_m_* (mM)**	1.83 ± 0.25	1.54 ± 0.13
***V_max_* (U/mg)**	312 ± 20	422 ± 44

## Abbreviation

ABTS	2,2′-azino-bis(3-ehylbenzothiazoline-6-sulfonic acid) diammonium salt
*C_A_*	Pharmaceutical concentration after removal
*C_B_*	Pharmaceutical concentration before removal
CBZ	Carbamazepine
DMF	*N*,*N*-dimethylformamide
HRP	Horseradish peroxidase
*I_A_*	Activity of free enzyme
*IM_A_*	Activity of immobilized enzyme
*k_d_*	Enzyme inactivation constant of free and immobilized HRP
*K_m_*	Michaelis-Menten constant
PVC	Poly(vinyl chloride)
*RA*	Enzyme relative activity
*RE*	Pharmaceutical removal efficiency
SA	Sodium alginate
SEM	Scanning electron microscopy
SMX	Sulfamethoxazole
THF	Tetrahydrofuran
*t*_1/2_	Enzyme half-life
*V_max_*	Maximum reaction rate
